# Evaluation of Existing Methods for Human Blood mRNA Isolation and Analysis for Large Studies

**DOI:** 10.1371/journal.pone.0161778

**Published:** 2016-08-30

**Authors:** Anke Meyer, Federico Paroni, Kathrin Günther, Gitanjali Dharmadhikari, Wolfgang Ahrens, Sørge Kelm, Kathrin Maedler

**Affiliations:** 1 Centre for Biomolecular Interactions Bremen, University of Bremen, Bremen, Germany; 2 Leibniz-Institute for Prevention Research and Epidemiology—BIPS, Bremen, Germany; 3 German Center for Diabetes Research (DZD) project partner, University of Bremen, Bremen, Germany; Naval Research Laboratory, UNITED STATES

## Abstract

**Aims:**

Prior to implementing gene expression analyses from blood to a larger cohort study, an evaluation to set up a reliable and reproducible method is mandatory but challenging due to the specific characteristics of the samples as well as their collection methods. In this pilot study we optimized a combination of blood sampling and RNA isolation methods and present reproducible gene expression results from human blood samples.

**Methods:**

The established PAXgene^TM^ blood collection method (Qiagen) was compared with the more recent Tempus^TM^ collection and storing system. RNA from blood samples collected by both systems was extracted on columns with the corresponding Norgen and PAX RNA extraction Kits. RNA quantity and quality was compared photometrically, with Ribogreen and by Real-Time PCR analyses of various reference genes (PPIA, β-ACTIN and TUBULIN) and exemplary of SIGLEC-7.

**Results:**

Combining different sampling methods and extraction kits caused strong variations in gene expression. The use of PAXgene^TM^ and Tempus^TM^ collection systems resulted in RNA of good quality and quantity for the respective RNA isolation system. No large inter-donor variations could be detected for both systems. However, it was not possible to extract sufficient RNA of good quality with the PAXgene^TM^ RNA extraction system from samples collected by Tempus^TM^ collection tubes. Comparing only the Norgen RNA extraction methods, RNA from blood collected either by the Tempus^TM^ or PAXgene^TM^ collection system delivered sufficient amount and quality of RNA, but the Tempus^TM^ collection delivered higher RNA concentration compared to the PAX^TM^ collection system. The established Pre-analytix PAXgene^TM^ RNA extraction system together with the PAXgene^TM^ blood collection system showed lowest C_T_-values, i.e. highest RNA concentration of good quality. Expression levels of all tested genes were stable and reproducible.

**Conclusions:**

This study confirms that it is not possible to mix or change sampling or extraction strategies during the same study because of large variations of RNA yield and expression levels.

## Introduction

A reliable analysis of gene expression is still challenging due to the various available blood sampling as well as subsequent RNA isolation methods, which result in robust changes of expression patterns. Nevertheless, gene expression analysis in peripheral blood is an important and obligatory tool in molecular research and diagnostics, especially in large epidemiological studies.

As a first major influencing aspect, sample collection and procession has a considerable influence on the data outcome. Consequently, for the pilot study presented here, the bio-sampling standards developed and evaluated during the IDEFICS (Identification and prevention of Dietary- and lifestyle-induced health EFfects In Children and infantS) study were transferred to this study to ensure reproducibility and comparability of the results [1]. The standardized survey procedures including instruments, methods, biological sampling and software tools as described in our previous work [2] were applied in this presented work.

Two major commercially available blood collection tube systems were available and compared in this study, (1) the PAXgene^TM^ and (2) the Tempus^TM^ collection tubes. The PAXgene^TM^ collection tubes (Preanalytix, Hombrechtikon, Switzerland) are loaded with a patented blend solution, which protects RNA molecules from degradation by RNases and prevents the induction of further gene expression. The Tempus^TM^ collection tubes (Life Technologies, Darmstadt, Germany) are preloaded with guanidine and detergent, which stabilizes the RNA by lysing the blood cells and inactivating the RNAses through chaotropic properties.

Previous analyses agreed that both PAXgene and Tempus systems cannot be combined in gene expression analyses because of large differences in RNA yields and expression levels [3–6]. A well-recognized first study on the topic compared the Tempus™ RNA stabilization reagent to PAXgene™ in leukemia patients [7]. The aim was to establish a routine protocol for blood sampling and isolation for later tests in patients with acute lymphoblastic leukemia (ALL) and chronic myeloproliferative leukemia (CML) and to compare the tube systems to non-stabilized EDTA blood. Besides the observation that RNA yields were considerably reduced with the Tempus™ system, the measured total gene expression varied in-between the tested sampling and storage methods [7]. In a second blood collection comparison, RNA was used for subsequent microarray analyzes [8]. It was described that both systems could be used for downstream applications [8]. In RNA isolated using the Life technologies “Tempus™ collection method”, *IFNγ*, *IL2*, *IL3*, *IL4*, and *IL13* is up-regulated in stimulated *vs*. control samples, which did not occur when the “PAXgene^TM^ collection method” was used [8]. This result clearly indicates that the reliability of the data is influenced by the RNA extraction method, and raises the demand for the application of one consistent workflow (including blood sampling tube). A comparison between results gained from different methods was not possible and there is no evidence, of which method results reflect the biological reality.

Also the next stages, i.e. RNA extraction and further quantification steps highly influence the results. In a previous microarray study, that used both collection systems, quantity and quality of RNA from three donors were comparable [9], but gene expression profiles clearly varied [9]. RNA isolated from both systems is of high quality, but the use of the Tempus RNA extraction system led to higher yield of nucleic acids, which was attributed to the decreased processing time in the Tempus RNA compared to PAXgene RNA extraction system [10]. Previous studies also found a correlation between temperature and RNA yield for the PAXgene RNA extraction [11], but not for the Tempus RNA extraction system [12].

Tempus^TM^ blood collection tubes yield higher amounts of RNA than PAXgene^TM^ [6]. The differences between the two systems may be explained either by suboptimal blood volume within the collection tubes or by their suboptimal shipping temperature. Particular attention must be paid to expression levels of immune system-related genes because of their rapid fluctuations. Microarray studies showed acceptable correlation between RNA from PAXgene^TM^ and Tempus^TM^ tubes [5, 13], however 443 genes mainly related to the immune system were differentially expressed in the two systems [5].

In contrast, Schramm *et al*. observed in samples from the KORA cohort, that different study designs or reagents (PAXgene™ vs. Tempus™ collection tubes) did not affect reproducibility in a microarray based analysis [13].

A further parameter, which strongly influences the RNA is the subsequent RNA extraction method. In this study, we compared several on column RNA extraction methods, which were specifically recommended by the manufacturers of the sampling systems. An additional criterion to setup the most appropriate method was the choice of a suitable reference gene for quantitative Real-Time PCR analysis of blood samples. Stable reference gene expression is one, if not the most critical, requirement for accurate and biologically meaningful gene expression normalization. Fluctuations of reference gene expression could lead to false down- or up-regulation of the target gene [14–16]. Here we provide a systematic analysis of the critical factors and their combination of blood sampling and subsequent RNA isolation and quantification.

## Material and Methods

### Blood collection

**Fig 1:**
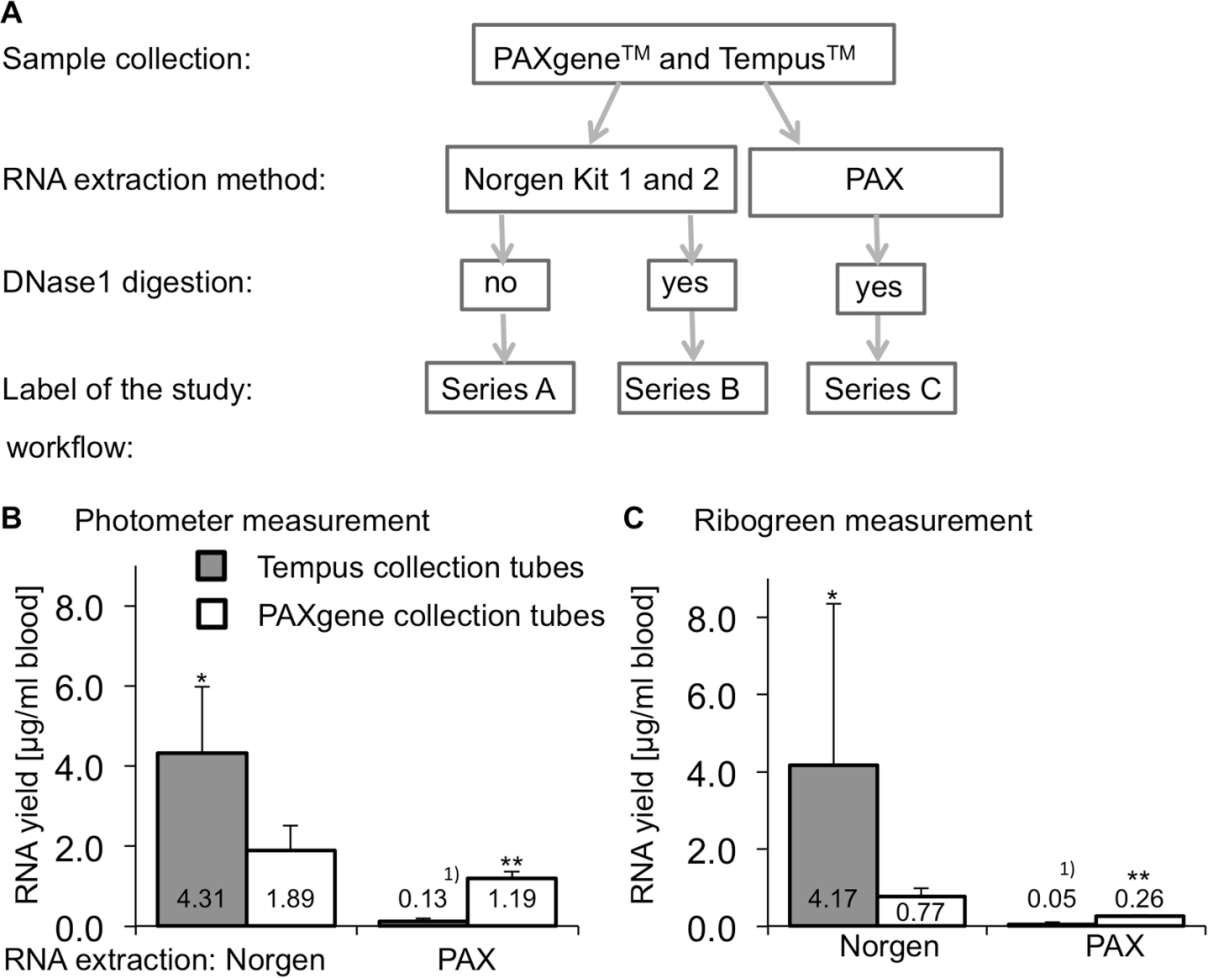
Study overview and RNA yields. **(A)** Study workflow of sample collection and RNA extraction. In the first sample set (n = 12 donors, 2 samples from each), all samples were extracted with the corresponding commercial Norgen Biotek kit, specified for both tested collection systems (series A&B). Series B was additionally DNase1 digested. A second sample set (series C; n = 5 donors, with 8 separately collected tubes from each donor for PAX and Tempus^TM^) was tested with the recommended PAXgene^TM^ blood RNA isolation kit. **(B)** RNA yield obtained with spectrophotometry (Nanodrop photometer) and **(C)** intact RNA yield measured with the Ribogreen method. ^1)^In 5 random samples no RNA was detectable. Therefore, those samples were excluded from calculations. *p<0.05 vs. PAXgene collection tubes/ Norgen RNA extraction, **p<0.05 vs. Tempus collection tubes/ Norgen RNA extraction and PAXgene collection tubes/ Norgen RNA extraction.

All 17 participants are voluntary test persons, of different age, sex and ethnical origin with confirmed good health status, good physical condition, free from medication and no current infectious disease. Two samples from each individual (n = 12) were collected at the same time by PAXgene^TM^ collection tubes (2.5 ml; Qiagen, Hombrechtikon, Switzerland; series A) and by Tempus^TM^ collection tubes (3.0 ml; ThermoFisher; series B; Fig 1A). In addition, eight samples from five individuals were collected (4 for PAXgene^TM^ = 2.5ml and 4 for Tempus^TM^ = 3.0ml) to compare the stability of the isolation (later named series C). All blood collection tubes were gently inverted for 5 min directly after collection and incubated for 2h at room temperature (RT), 48h at 4°C and then stored at -80°C until processing. Samples were thawed on ice and left another 2h at RT before RNA extraction. Ethical approval for the study was obtained by the Ethical Committee of University of Bremen. The participants of the study provided their written informed consent to participate in this study. The ethics committee of University of Bremen approved this consent procedure.

### RNA extraction

During the test period, only the corresponding RNA extraction kit for PAXgene^TM^ tubes was commercially available. Therefore, in the first sample set (n = 12), RNA was extracted with a commercial kit from Norgen Biotek (Norgen, Thorold, ON, Canada), specified for both tested PAX (Norgen Biotek RNA purification Kit II) and Tempus^TM^ (Norgen Biotek RNA purification Kit I) collection systems, referred to as *Norgen RNA extraction kit* throughout the manuscript. A second sample set (series C, n = 40, 5 donors 8 tubes each donor for PAX (n = 4) and Tempus (n = 4)) was tested with the recommended PAXgene^TM^ Blood RNA isolation kit, referred to as *PAXgene RNA extraction kit*. All extractions were performed according to the manufacturers’ protocols as described below.

#### Norgen Biotek RNA purification Kit I (used with Tempus^TM^ tubes)

Blood samples were transferred to a 50 ml tube and filled with Tempus^TM^ Blood RNA tube diluent to a final volume of 12 ml. Tubes were vortexed vigorously for 30 s. Mixed samples were centrifuged at 4°C for 30 min at 3,000xg. The dried pellet was re-suspended with 600 μl lysis solution by vortexing. 300 μl 96% Ethanol was applied to the samples and mixed. Afterwards, the solutions were transferred to the RNA isolation spin columns. Centrifugation for 1 min at 14,000xg was performed before samples were washed with washing buffer and for the optional DNase 1 digestion, 100 μl RNase-free DNase 1 was applied to the sample, centrifuged for 1 min at 14,000xg and flow through added to the column again. DNA digestion was carried out at RT for 15 min. Thereafter, 400 μl of the washing solution was added and the columns centrifuged for 1 min at 14,000xg and then for 2 min at 16,900xg to dry the column membrane. RNA was eluted by adding 50 μl elution buffer to the samples and incubated for 1 min followed by centrifugation for 2 min at 200xg and 1 min at 14,000xg. The RNA elution step was repeated.

#### Norgen Biotek RNA purification Kit II (used with PAXgene™ tubes)

PAXgene^TM^ tubes were centrifuged for 10 min at 5,000xg. After decanting the supernatant, the pellet was liquefied with 4 ml NPX1 and vortexed until the pellet was dissolved. The tubes were centrifuged again for 10 min at 5,000xg. In the next step, the supernatant was discarded and the pellet was lysed in 600 μl NPX2 by vortexing. Centrifugation was carried out to remove insoluble materials for 1 min at 14,000xg. The supernatants were transferred to new tubes and mixed with 300 μl 96% ethanol by vortexing. The solutions were transferred to the RNA isolation spin columns. Centrifugation for 1 min at 14,000xg was performed before samples were washed with 400 μl NPX3 and centrifuged for 1 min at 14,000xg. Optional DNase 1 digestion was performed as described above. Thereafter, 400 μl of NPX3 was added and columns were centrifuged for 1 min at 14,000xg. Washing with 400 μl NPX3 was repeated and then 2 min of centrifugation at maximum speed was applied to dry the column membrane. The RNA was eluted to the provided storing tubes by addition of 50 μl NPX5 to each sample and incubated for 1 min followed by a centrifugation for 2 min at 200xg and for 1 min at 14,000xg. The RNA elution step was repeated.

An additional DNase1 digestion was performed for series B (Norgen extraction systems). Here 250ng total RNA were digested in three independent experiments with DNase 1 ([1U], Fermentas, St. Leon-Rot, Germany). All the samples were used later for cDNA synthesis in at least technical duplicates (qRT-PCR) [17].

#### PAXgene™ RNA isolation Kit

Tubes were centrifuged for 10 min at 3,000xg. After decanting the supernatant, the dried tubes were refilled with 4 ml RNase free water and pellets re-suspended by vortexing. Thereafter, a next centrifugation step for 10 min at 3,000xg was performed. The pellet was re-suspended in 350 μl BR1 and transferred to a 2 ml tube. The observation was made that samples from Tempus^TM^ tubes did not resolve well in BR1 buffer. Hence, samples were mixed with 300 μl BR2 and 40 μl Proteinase K followed by 10 min incubation at 55°C at a shaker. Samples were pipetted to QIAshredder columns and tubes centrifuged 3 min at 17,000xg. The transferred supernatant of the flow through was mixed with 700 μl 100% Isopropanol. Samples were stored at -80°C until the next step was performed.

Samples were equilibrated to RT, placed in the RNA isolation column and centrifuged for 1 min at 12,000xg. Samples were digested with DNase1 on-column; 350 μl BR3 was used to equilibrate the column conditions before adding 80 μl DNase RDD mix. Following 15 min incubation at RT, the samples were washed with 350 μl BR3. Samples were treated twice with 500 μl BR4 washing buffer and centrifuged 1 min at 12,000xg. A final centrifugation step was performed for 3 min at 17,000xg to dry the membrane. To elute the RNA, 40 μl BR5 were supplied to the membrane, incubated for 1 min and centrifuged 1 min at 12,000xg; the elution step was repeated. For denaturation, the RNA was incubated for 5 min at 65°C in a heating block.

### RNA validation

Integrity and quality of isolated RNA was measured by the classical absorbance measurement at 260 nm by the NanoDrop™ spectrophotometer (“photometer method”; Nanodrop, Thermo Scientific, Schwerte, Germany) and by RiboGreen fluorescence measurements, performed in a volume of 200 μl and fluorescence emission measured at 520 nm against a standard reference curve according to the manufacturer’s instructions. The RiboGreen^®^ reagent is an ultra sensitive fluorescent nucleic acid stain for quantifying RNA in solution without the absorbance method-based disadvantages such as signals from contaminating proteins or interference of free nucleotides.

### cDNA synthesis

Reverse transcription reactions were performed in a volume of 20 μl containing 500 ng (series A), 250 ng (series B) or 100 ng (series C) of total RNA (Fig 1A). First strand cDNA synthesis was carried out with 200 ng random hexamers (Fermentas, St. Leon-Rot, Germany). The sample and primer mix was denatured in 5 min at 70°C and subsequently chilled on ice. Incubation with 5X reaction buffer, dNTPs (1 mM) and 200 Units (U) Reverted Aid reverse transcriptase (Fermentas, St. Leon-Rot, Germany) was performed at 25°C for 10 min followed by 60 min at 42°C and 10 min at 70°C.

### Real Time PCR

Real-time RT-PCR was performed using hydrolysis probes and the 2x Taqman Universal PCR Mastermix with an ABI Stepone Plus Cycler (Applied Biosystems, Darmstadt, Germany). Reactions were performed in duplicates in a volume of 10 μl with specific primers and probes (*SIGLEC7* Hs00255574_m1 (exon spanning probe; PCR efficiency 95%), *PPIA* Hs99999904_m1 PCR efficiency 100%, β-Actin Hs99999903_m1 (exon spanning probe; PCR efficiency 94%), Tubulin Hs00362387_m1 (exon spanning probe; PCR efficiency 99%), all Applied Biosystems, Darmstadt, Germany). Primer efficiencies were tested in dilution series of a single exemplary investigated blood sample. For all amplicons, a cDNA component control and a control without reverse transcriptase were performed. Cycling program: 20 s 95°C and 50 Cycles: Denaturation 1 s 95°C followed by 20 s 60°C annealing and elongation. Results are presented as C_T_ or RQ to a calibrator (ΔC_T_) [18] (StepOne Software Version 2.2.2). Real-Time PCR data were efficiency corrected and normalized either to individual reference or to mean reference gene expression [19].

### Statistics

RT-PCR results were calculated according to the ΔΔCt method and corrected on single gene efficiency to minimize error propagation [20]. The results presented are means ± standard deviations (SD) of individual RNA values or relative quantification. Fold of change are presented in Log_2_ scale for symmetry improvement. The significance of difference between individual experiments was tested by student’s t-test. P values of p<0.05 was considered to be statistically significant.

## Results

### RNA quality and quantity highly depends on collection system and extraction method

System-specified amounts of blood were collected by PAXgene^TM^ (2.5 ml) and Tempus^TM^ (3.0 ml) collection systems (series A + B). RNA of both was isolated either by the Norgen 1 (specific for Tempus^TM^) and 2 (specific for PAX) or the PAX RNA isolation systems. An additional DNase1 digestion for both tubing systems was performed to prevent any possible genomic contamination (Fig 1A).

The RNA yield was adjusted to the respective kit specified sample and elution volume (Fig 1B and 1C) and final intact RNA concentration measured by Nanodrop spectrophotometer and the Quant-iT™ RiboGreen^®^ RNA kit, to exclude the problem of contaminating proteins and free nucleotides in solution (degraded nucleic acids).

High purity RNA was obtained from both PAXgene and Norgen RNA extraction kits with average E_260_/E_280_ ratios of Tempus™ 2.1±0.01 *vs*. PAXgene™ 2.0±0.03, irrespective of the blood collection system. An exception is the RNA from Tempus^TM^ collection tubes extracted with the PAXgene RNA extraction kit. Here, five samples from various series showed no RNA and the remaining samples showed a high mean E_260_/E_280_ ratio of 2.5±1.26 and were omitted from further analysis.

RNA extracted with the Norgen RNA extraction kits from Tempus™ collection tubes showed almost identical concentrations with both analysis methods. In contrast, 2.5-fold higher RNA values were obtained with the photometer from the PAXgene™ collection tubes, compared to Ribogreen (Fig 1B and 1C). This shows that significant differences in RNA amount could be revealed from RNA extractions from different collection tubes. Using the Norgen RNA extraction kit, Tempus collection tubes showed a higher RNA yield, compared to the PAX collection tubes (2.3-fold by photometer, 5.4-fold by Ribogreen; Fig 1B and 1C).

Together with the low RNA quality, RNA extracted with the PAXgene RNA extraction kit from Tempus™ collection tubes showed the lowest yield with almost no detectable RNA. In contrast, the RNA yields from PAXgene RNA extraction kits were improved when compared to the PAXgene collection tubes, but were much lower than those from both Norgen RNA extractions kits (73 and 94% reduction compared to Tempus collection tubes/ Norgen RNA extraction; 37 and 66% to PAXgene collection tubes/ Norgen RNA extraction by photometer (Fig 1B) and Ribogreen (Fig 1C)).

Surprisingly, the average intact RNA yield from PAXgene™ collection tubes was higher with the Norgen RNA isolation kit than with the PAXgene™ kit (2.9-times higher by Nanodrop and 1.6-times higher by Quant-iT™ RiboGreen^®^ measurements).

In summary, the combination of Tempus^TM^ blood collection tubes and Norgen RNA extraction delivered the highest RNA yields. But the RNA yield variation was highest from the Tempus^TM^ blood collection tubes, while PAX collection-PAX extraction showed lowest variations (referring to a maximum difference of 100 ng/ml blood).

### *SIGLEC-7* gene expression is highly influenced by the sampling system, isolation method and reference genes

#### Higher mRNA expression levels by Norgen mRNA isolations from Tempus blood collection, compared to PAX collection tubes

To further characterize the RNA quality despite the differences in yield, RT-PCR analysis was performed on several reference genes as well as on *SIGLEC-7*, an adhesion molecule expressed on monocytes [21] with high individual variation, which we have reported previously to regulate pancreatic β-cell survival and immune cell activation in diabetes [22]. Different *SIGLEC-7* gene expression levels among individuals dependent on their status of health and inflammation were observed in our laboratory before [23]. This was confirmed here; absolute changes did not only occur in relation to the sampling systems, extraction methods and normalization strategies, but also in a donor-dependent manner.

**Fig 2:**
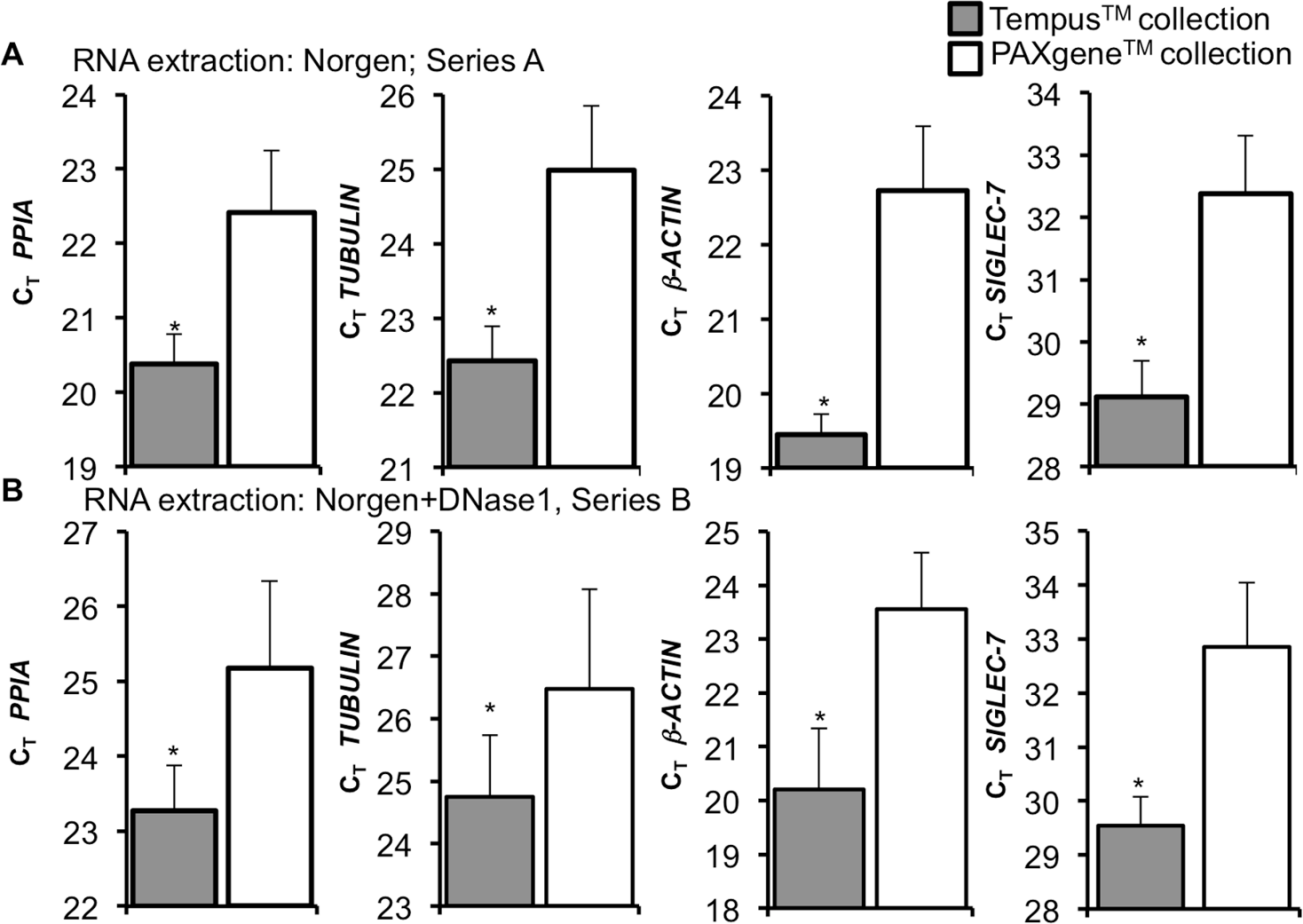
Comparison of the C_T_-values generated by Series A&B. Real-Time PCR quantification of blood samples collected with Tempus^TM^ and PAXgene^TM^ collection tubes and RNA extracted by Norgen. **(A)** Series A: Norgen RNA extraction with 500 ng RNA input (n = 12 each for Tempus and for PAXgene blood collection), **(B)** Series B: Norgen RNA extraction and a second DNase1 digestion with 250 ng input RNA (n = 12 each for Tempus^TM^ and PAXgene^TM^ collection tubes, experimental triplicates from DNA digestion onwards). Data are means of all donors ± SE *p<0.05 vs. PAXgene collection tubes.

Since Norgen RNA extraction delivered the highest RNA yields, this kit was used for further analyses of the same samples of Tempus and PAX collected blood (Fig 2A and 2B). Samples were split into two groups; Series A (Figs 1A and 2A); and Series B with an additional DNase1 digestion (Figs 1A and 2B). This more reliable DNA digestion setup was necessary, because in series A the negative control without reverse transcriptase resulted in values for *PPIA*, suggestive of DNA contamination in the RNA, although an on-column-DNA-digestion had been performed within the Norgen kit.

In line with the highest RNA yields (Fig 1), samples collected from Tempus blood collection tubes showed lower C_T_ values throughout series A and B, compared to PAX collection tubes (Fig 2A and 2B). Variations were detectable and similar in all tested genes in both series. However, Series A showed lower C_T_ values and variability compared to series B probably due to the effect of traces of genomic contamination (Fig 2A and 2B).

#### Normalization of a pool of reference genes delivers more reliable results

**Fig 3:**
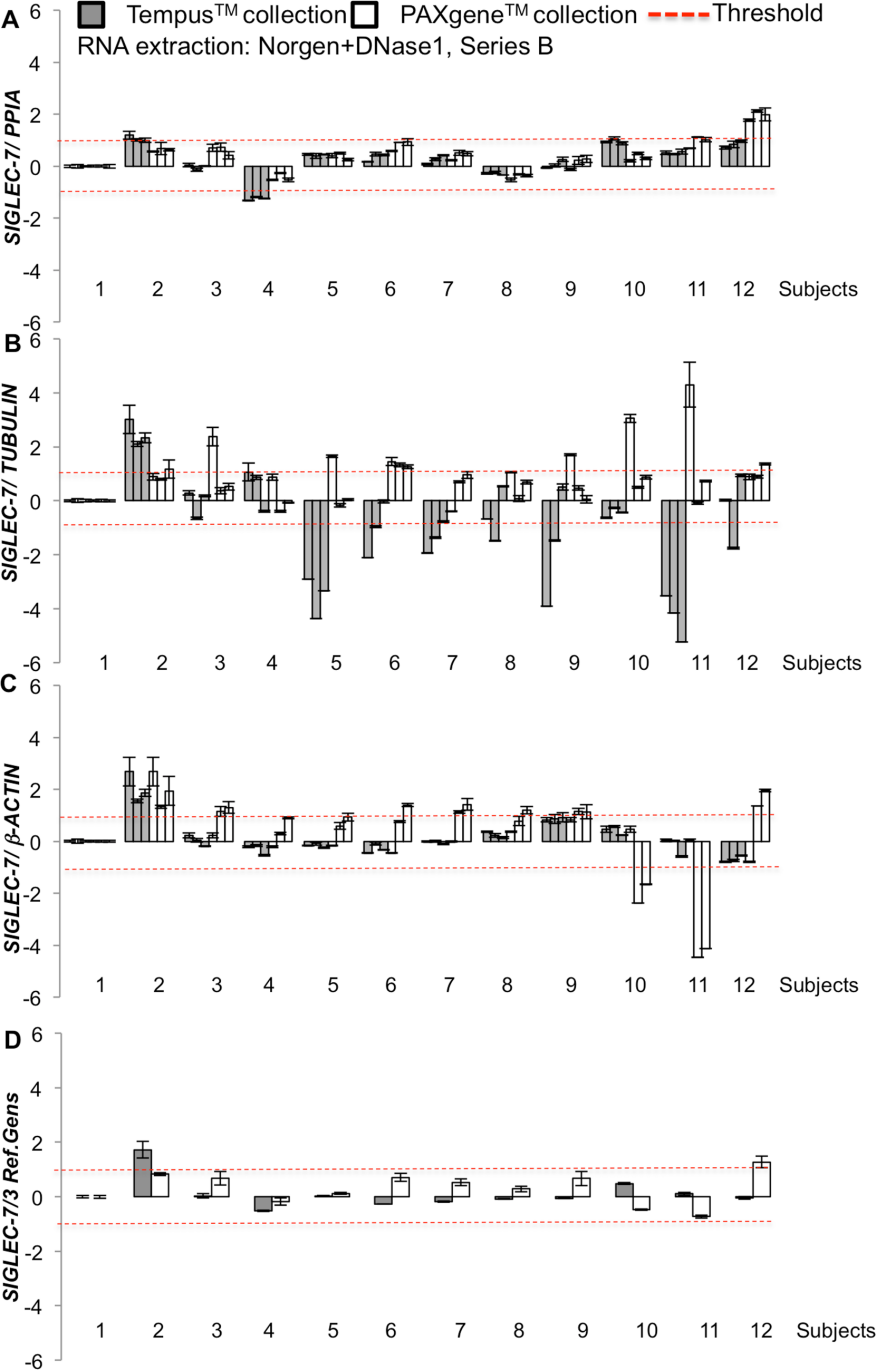
RT-PCR analysis results are affected by sampling system, RNA extraction method and the choice of reference gene. *SIGLEC-7* gene expression profiles of Series B with additional DNAse digestion. Blood was collected from 12 donors; triplicates from each donor were reverse transcribed and experimental duplicates from each sample run in Real-Time PCR reactions. Data were normalized to **(A)** *PPIA*, **(B)** *β-ACTIN*, **(C)** *β-TUBULIN* and (**D**) to a combination of these 3 reference genes. Results are shown as relative to the randomly chosen subject #1. A-C show means of experimental duplicates ± SE, D shows means of pooled triplicates from each donor.

To proof the assay sensitivity, an additional series B was generated, in which a lower amount of 250 ng RNA was digested and subsequently reverse transcribed. DNase1 digestion and RT-PCR was performed in 3 sets of replicates from each sample and ran on different days (Fig 3). No DNA based contamination could be detected (data not shown). Here we could show relative comparison of gene expression levels normalized on 3 reference genes (Fig 3A–3C) as well as the combination of the 3 (Fig 3D). In order to achieve this comparison, mRNA expression levels were normalized on one random subject (#1). When *PPIA* was used as reference gene, both data sets from Tempus and PAXgene collection tubes showed a high inter and intra assay reproducibility among the single donors, compared to the other reference genes (Fig 3A). These results were also independent of the yield of RNA achieved from the two systems (Figs 1 and 2). In contrast, the normalization on *TUBULIN* and *β-ACTIN* resulted in high fluctuation of the results with even opposite results from the 2 collection systems (e.g. in subjects 5–11 for TUBULIN). Normalization on 3 reference genes resulted in an upregulation above the set arbitrary threshold of 1 (which is sufficient to confidentially distinguish a log2 2-fold of change or 1 cycle-difference) only in subject #2, confirming the upregulation, which also occurred independently in all 3 separate reference gene normalizations.

#### Stable mRNA expression levels by PAXgene mRNA isolations only from PAX collection tubes

**Fig 4:**
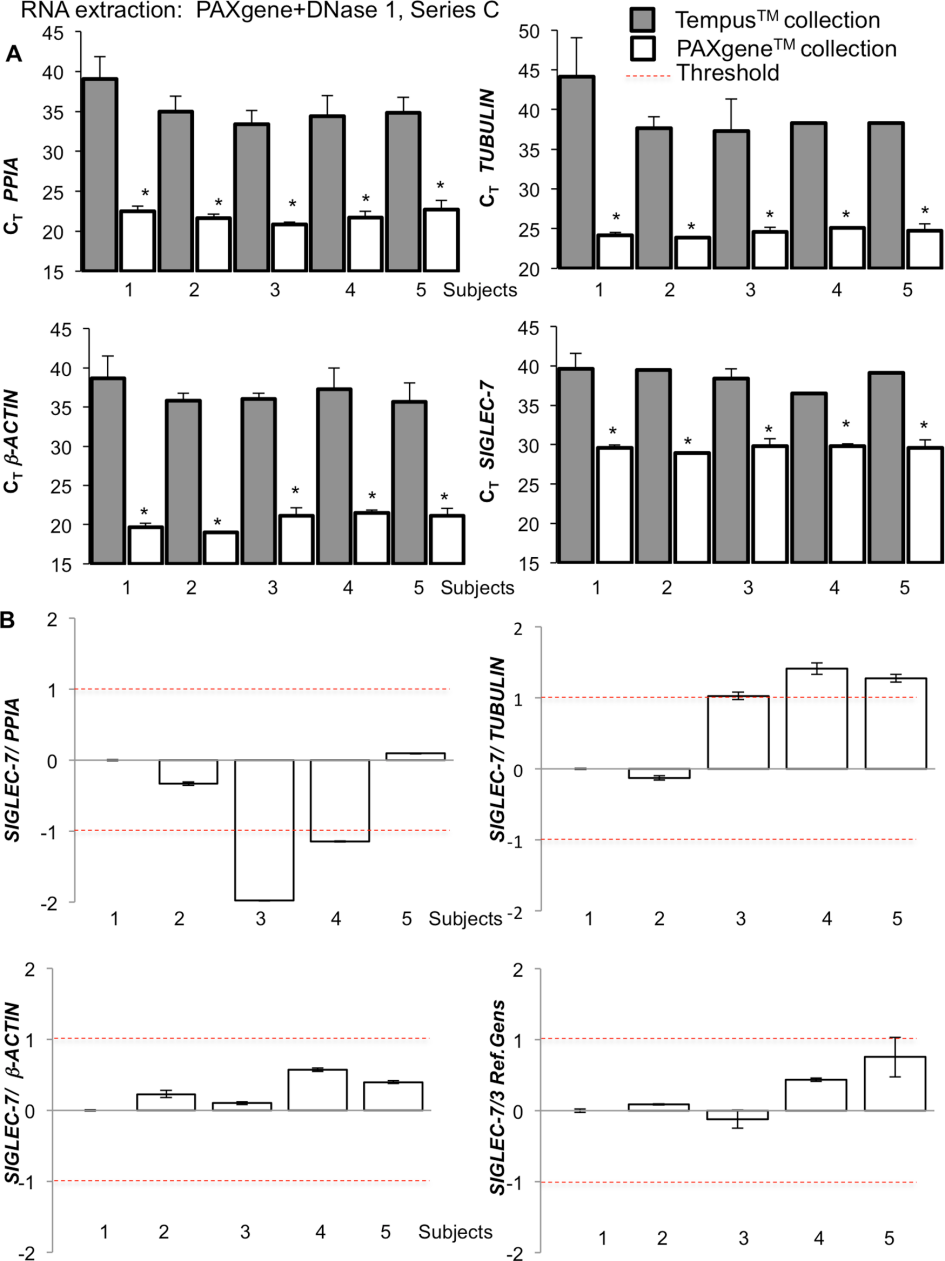
Comparison of the C_T_-values generated by Series C. **(A)** Real-Time PCR quantification of blood samples collected with Tempus^TM^ and PAXgene^TM^ collection tubes and RNA extracted by PAXgene with a second DNase1 digestion with 100 ng input RNA (n = 5; 4 separately collected samples from each participant). **(B)** *SIGLEC-7* gene expression profiles of Series C. Data were normalized to *PPIA*, *β-ACTIN*, *β-TUBULIN* and to a combination of these 3 reference genes. Results are shown as relative to the randomly chosen subject #1. Data are means of 4 separately collected samples ± SE. *p<0.05 vs. TEMPUS collection tubes.

Next, the intra donor stability of gene expression was tested in blood RNA samples collected from the same donor in parallel (series C). Four tubes PAXgene^TM^ and Tempus^TM^, respectively, were collected from 5 donors and RNA amount and gene expression compared. As expected from the very low RNA yield (Fig 1C), gene expression from Tempus^TM^ collection tubes/ PAXgene^TM^ RNA isolation showed high C_T_ values (C_T_>33; Fig 4A). Therefore, the relative quantification comparison is shown only with the samples from PAXgene^TM^ collection tubes (Fig 4B). Stability evaluation of *SIGLEC-7* expression was performed in a setting of samples from 5 donors by PAXgene tube collection, PAXgene RNA extractions (series C) in quadruplicate (4 seperate tubes) and subsequent PCR analysis). Data were normalized to *PPIA*, *β-ACTIN*, *TUBULIN* and their combination (Fig 4B).

In accordance with the data from Norgen isolation (Fig 3), *SIGLEC-7* gene expression results strongly differed dependent on the normalization to different reference genes (Fig 4B). The lowest individual *SIGLEC-7* gene expression variations from one single donor occurred when normalized to *PPIA* (Fig 3A). As before, comparison of changes in *SIGLEC-7* gene expression levels among the donors showed fluctuations in gene expression, spanning from downregulation to upregulation, dependent on the choice of the normalization gene (e.g. subject 4 was downregulated when normalized on PPIA, uregulated on *TUBULIN* or stable on *β-ACTIN*). Interestingly, such fluctuation was abrogated when *SIGLEC-7* expression was normalized on 3 reference genes. These analyses make the normalization to the pool of reference genes indispensible in order to get reliable expression data.

In summary, the exemplary chosen *SIGLEC-7* gene expression showed high variations (1) with different sampling systems, (2) with different extraction methods and (3) with normalization on different reference genes. The question remains, which system is the one of choice and results in most stable expression levels.

**Fig 5:**
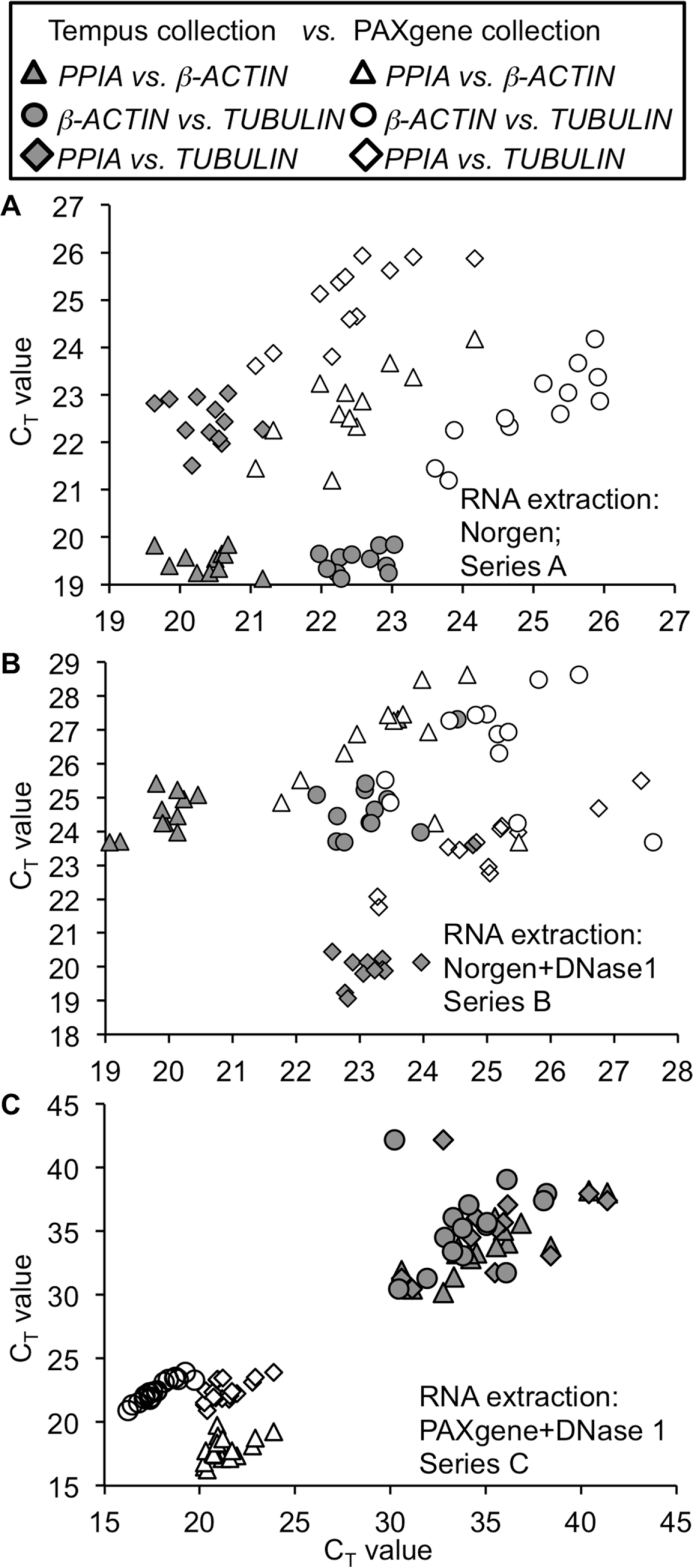
Coupling of PAX collection tubes with PAXgene RNA extraction kit improved RNA stability, quantity and quality and enhanced reproducibility. Plotted dots of donors 1–12 from different reference C_T_ values from the analyses in Figs 2 and 4 (Series A,B,C). Results from Fig 2 were used and C_T_ values from house keeping genes were compared from the 12 different donors. Data are obtained **(A)** from series A with 500 ng RNA (n = 12; both sampling systems), **(B)** from series B with 250 ng RNA with additional DNase1 digestion and (**C)** 5 donors with 4 samples each from Tempus^TM^ and PAXgene^TM^ collection (100 ng each measured in experimental duplicates).

To address this, we plotted all results with its various determinants from Fig 2 (Tempus and PAXgene blood collection/Norgen RNA extraction; Series A&B) and Fig 4 (PAXgene blood collection/PAXgene RNA extraction) in one single graph for each of the Series A, B and C (Fig 5). From an ideal analysis we would expect that same C_T_ values of reference genes are obtained from all donors when using the same RNA concentration. As shown in Fig 5A and 5B (Series A and B), Tempus^TM^ collection tubes in combination with Norgen isolation kit delivered higher reproducibility, compared to Pax/Norgen. Nevertheless the coupling of PAX collection tubes with PAXgene^TM^ isolation kit (Series C; Fig 4C) further improved RNA stability, quantity and quality together with enhanced reproducibility.

## Discussion

For gene analysis profiles from large epidemiological studies it is essential to generate a stable handling protocol, which starts with the blood collection and storage system. It is well known that RNA is sensitive to degradation by postmortem processes; inadequate sample handling or storage. Especially in blood samples, degradation processes start early because of the existence of high levels of RNAses. Hemoglobin in whole blood can lead to clogging of extraction matrices, which makes blood RNA isolation further difficult and gene expression analyses unreliable. For example, high expression changes of genes for interleukins or interferons are observed directly after blood donation [8]. To account for such problems, specific blood collection and subsequent RNA isolation systems were developed by several manufacturers and compared in this study. Gene expression levels from full blood show high variations, which result from the use of different blood collection and RNA extraction systems as well as from the normalization to different reference genes. Such variations do not allow any comparison of RNA obtained from different blood sampling or RNA isolation protocols.

Other factors, e.g. high globin mRNA present in red blood cells also interfere with gene expression profiling analyses, which may be especially problematic in microarray analyses [4]. In both PAXgenes and Tempus systems, globin depleted RNA showed 60% more transcripts than samples with higher globin mRNA. Importantly, enzymatic globin depletion reduces RNA quality [5], and thus methods of non-enzymatic depletion have to be used [4, 5]. However, RiboAmp mRNA amplification in a microarray study did not amplify globin mRNA and thus, additional globin clearance was not found necessary in this previous study [5].

Here, *SIGLEC-7* mRNA, which is mainly expressed on monocytes [21], was analyzed in whole blood RNA samples. We aimed to set our comparison on such gene, which is rather low-expressed in whole blood samples, since small expression changes would be visualized in large variations. A donor dependent comparison of *SIGLEC-7* does not exist yet, and we aimed to see whether we could get sufficient *SIGLEC-7* signals from full blood, which represents monocytic expression. We found reproducible and comparable signals from whole blood extracts, and conclude that *SIGLEC-7* analyses can be included in large population studies without the necessity to purify monocytes from small amounts of blood.

The comparison of various available methods shows that the most consistent results were achieved with the combination of PAXgene^TM^ blood collection together with the PAXgene^TM^ isolation system (our Series C). The combined PAXgene^TM^ system presented the highest gene expression as well as most stable results. In contrast, RNA extraction from Tempus^TM^ collection tubes combined with PAXgene^TM^ blood isolation kit delivered an extremely low RNA yield and such combination is therefore not advisable.

To assess whether PAXgene™ and Tempus™ peripheral blood RNA collection tubes provide higher quality and RNA yield, we compared their downstream application by RT-PCR; stable RT-PCR results from the same samples would allow evaluation of the RNA quality. Based on such RNA quality and quantity measurements, Tempus™ blood collection tubes combined with Norgen RNA isolation delivered the highest amount of RNA with comparable quality.

Our results are consistent with Duale et al [6] and Hantzsch et al [3], who also achieved higher RNA concentrations from blood collected with Tempus tubes, compared to those from PAXgene. Also it was reported that RNA extraction from the PAXgene blood collection using the Norgen RNA kits delivered higher C_T_ values [3], which was also seen in our study (compare Figs 2 and 4).

In contrast, the combination of Tempus™ tubes with PAXgene™ RNA extraction delivered low amounts of RNA. The yield of RNA recovery has been very poor and closed to no RNA was obtained. Such problem becomes clear by looking at the stabilizing agent used in the two kits. Tempus^TM^ collection tubes are based on guanidinium hydrochloride as RNA stabilizing agent while PAXgene^TM^ collection tubes use a chemistry of tetradecyl-trimethylammonium oxalate buffered with tartaric acid [24]. Tempus^TM^ blood collection tubes and RNA extraction kits are designed to extract RNA within an alkaline pH range (>8); oppositely to PAXgene^TM^ blood collection tubes and RNA extraction kit which are performed within an acidic pH range of 3–4. After the first centrifugation step, pellets in acid (PAXgene^TM^) and alkaline (Tempus^TM^) pH are generated, respectively. Resuspension of the Tempus^TM^ pellet with PAXgene^TM^ blood RNA extraction kit solution would then increase the pH of the solution affecting binding of the nucleic acid to the column matrix, which has also been optimized for a pH of 3–4 [25]. Therefore, Tempus tubes are not suitable for PAXgene^TM^ blood RNA extraction kit by their nature. Thus, only RNA from PAXgene™ tube samples could be processed for the downstream RT-PCR analyses. Such PAXgene™ extracted RNA from PAXgene™ (Series C) collection tubes showed higher RNA expression levels by C_T_ values together with improved quality of the Real-Time PCR reaction than series B (Tempus-Norgen). Even with a lower total amount of extracted RNA, the use of the PAXgene extraction kits in the Real-Time PCR resulted in a much better sensitivity of around 3 C_T_ values lower and improved signals with less than half of the initial RNA amount.

The downstream application of series A and B (Norgen extraction) in Real-Time PCRs showed a more constant signal with the RNA obtained from Tempus™ tubes, compared with those blood samples from the PAXgene^TM^ collection. But only transcripts ≥200 bp can be isolated with the Tempus™ kit [7], while RNA extraction with the PAXgene™ isolation kit allows RNA transcripts down to 50 bp [7], which could lead to discrepancy in RT-PCR results between the two kits.

DNA traces were detected in all blood samples from PAXgene™ and Tempus™ tubes independent of the use of the Norgen or PAXgene^TM^ RNA isolation kits and the solely on-column DNAse digestion was not sufficient for a complete DNA-contamination removal. Used primers in this study span exon-exon boundaries, except *PPIA* primers, where the amplicon spans an exon junction and the probe and one of the primers sit within one exon, thus, this assay may be affected from the presence of genomic DNA. Even for primers spanning exon-exon boundaries, DNAse digestion would result in more reliable analyses, because large amounts of remaining DNA possibly disturb the primer annealing during the PCR reaction.

The total amount of RNA extracted from the PAXgene™ collection system was higher when isolated with the Norgen instead of the PAXgene™ RNA isolation kit. But as already said above, downstream application of RT-PCR shows that PAXgene^TM^ extraction from PAXgene^TM^ collection delivered lower C_T_ values, i.e. higher gene expression, even when the reaction was performed with a lower starting RNA amount. This suggests that the overall RNA integrity is enhanced using the PAXgene^TM^ system.

A previous work demonstrated an approach to minimize systematic errors by the use of multiple reference genes as normalization strategy [19]. Still, and also suggested by our study, an initial step to confirm consistency of reference genes is mandatory [19]. In our approach, we provide the comparison of different blood sampling and RNA isolation methods and the relative comparison of gene expression levels, normalized on three independent reference genes as well as on their combination. Three different classical reference genes could result in opposite expression level changes for both Tempus and PAXgene^TM^ blood collection systems. *PPIA* showed the lowest inter- and intra-assay variability compared to both *β-Actin* and *β-Tubulin*. The gene of interest used in the present study (*SIGLEC-7*) showed high C_T_ values (around 30), which, in particular in blood samples, is associated with higher expression variability. A possible approach is to narrow down the confidence interval with an increase of replicates, but this could often be limited by the availability of samples. To avoid such gene expression uncertainty, we show here that the use of a combination of three not closely related housekeeping genes represents an option of choice to avoid false positives (either up- or downregulated), particularly when genes with known high variability are under scrutiny. Also position of primers and probes, amplicon length as well as RNA’s secondary structures can further affect gene expression levels detected by RT-PCR. In our case *β-ACTIN* and *β-TUBULIN* probes bind to the 5’-mRNA region (50–300bp) while *PPIA* and *SIGLEC-7* probes anneal around 1kb from the 5’-end.

In parallel with the higher RNA amount, genes analyzed in series A and B (Norgen extraction) showed generally higher C_T_-values/equal amount of RNA from samples collected in the PAXgene™ tubes. Still, the distribution of the plotted C_T_ values was different between the systems. A more linear correlation was obtained with samples generated from PAXgene™ tubes, which confirms the larger variation in gene expression within the samples. In contrast, Tempus tube collection with Norgen RNA extraction (Series A and B) delivered more stable results with the values assembled in a small cloud. Yet, in series C, constant expression levels of the reference genes were measured from the PAXgene system. The smaller the variance the higher was the reliability of the reference gene expression and thus the overall data trustworthiness.

Whole blood is the easiest obtained and therefore most used human material, which can be used to deduce information about the health state of the donor. With the data determined in this investigation, the important knowledge of “how my RNA was collected”, “how to proceed with the RNA” as well as the importance of the choice of the reference gene are highlighted. In general, both tubing systems deliver RNA in good quality and amount, but random combinations with the RNA extraction systems must be avoided.
